# Continuous Attractor Neural Networks: Candidate of a Canonical Model for Neural Information Representation

**DOI:** 10.12688/f1000research.7387.1

**Published:** 2016-02-10

**Authors:** Si Wu, K Y Michael Wong, C C Alan Fung, Yuanyuan Mi, Wenhao Zhang

**Affiliations:** 1 State Key Laboratory of Cognitive Neuroscience & Learning, IDG/McGovern Institute for Brain Research, Beijing Normal University, Beijing, 100875, China; 2 Department of Physics, Hong Kong University of Science & Technology, Clear Water Bay Peninsula, Hong Kong; 3 RIKEN Brain Science Institute, Wako-shi, Saitama, Japan

**Keywords:** Continuous Attractor Neural Network, Neural network, multi-sensory information integration, anticipative tracking, canonical model

## Abstract

Owing to its many computationally desirable properties, the model of continuous attractor neural networks (CANNs) has been successfully applied to describe the encoding of simple continuous features in neural systems, such as orientation, moving direction, head direction, and spatial location of objects. Recent experimental and computational studies revealed that complex features of external inputs may also be encoded by low-dimensional CANNs embedded in the high-dimensional space of neural population activity. The new experimental data also confirmed the existence of the M-shaped correlation between neuronal responses, which is a correlation structure associated with the unique dynamics of CANNs. This body of evidence, which is reviewed in this report, suggests that CANNs may serve as a canonical model for neural information representation.

## Introduction

The brain performs computation via dynamics of neural circuits formed by a large number of neurons. The dynamics of a neural circuit, on the other hand, are determined by the connection pattern between neurons. Thus, unveiling the structures of neural networks and their associated dynamical properties is at the core of elucidating brain functions. A question of common interest in both experimental and computational neuroscience is whether there exist canonical circuit models for neural information processing.

Over the past few decades, a type of recurrent network, known as the continuous attractor neural network (CANN) or dynamic neural field, has received broad attention from computational neuroscientists ^1– 3^. This model has been successfully applied to describe the encoding of continuous stimuli in neural systems, such as orientation ^4^, moving direction ^5^, head direction ^6^, and spatial location of objects ^7^. The model has many computationally appealing properties, such as efficient population decoding ^8^, smooth tracking of moving objects ^9^, and implementing parametrical working memory ^10, 11^. The computational advantages of CANNs and their successes in modeling brain functions have suggested that CANNs serve as a canonical model for neural information representation. While there has been some evidence for CANN characteristics in the brain (e.g. the movement map in the superior colliculus) ^12^, we review here recent important experimental findings and some new results for applying CANNs to modeling brain functions.

## The model of CANNs

**Figure 1.**
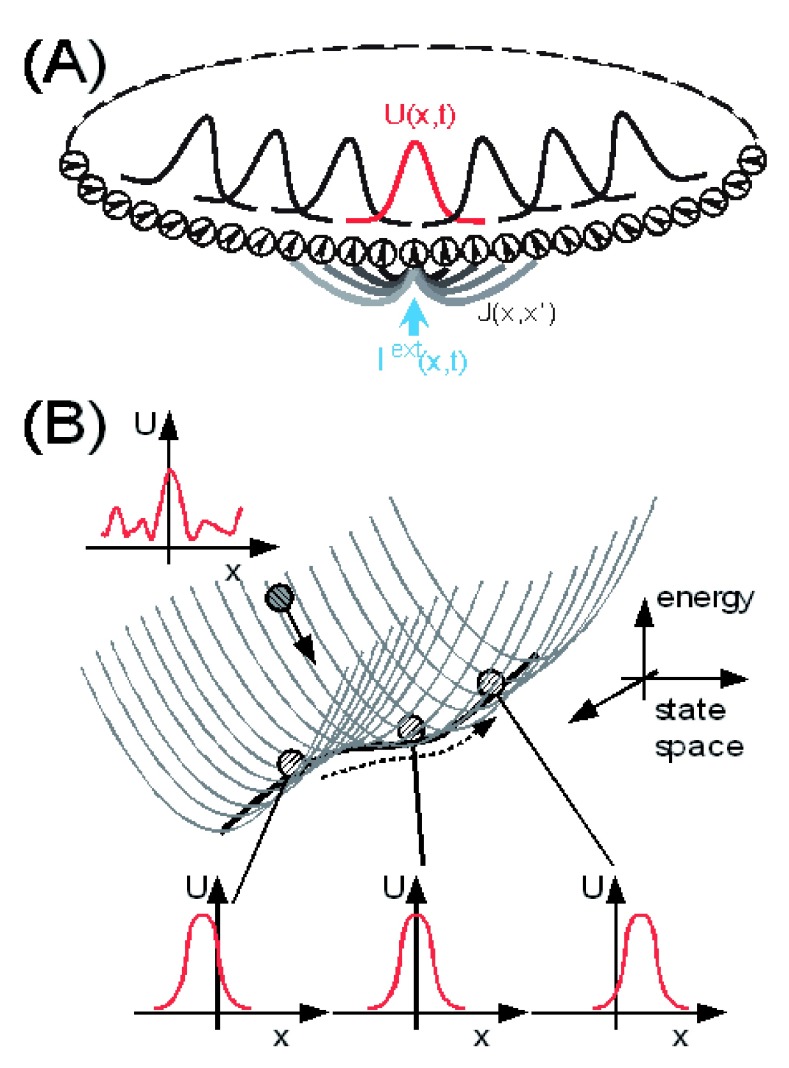
A continuous attractor neural network (CANN) model. ( **A**) An illustration of a one-dimensional CANN, which encodes a continuous variable (e.g. orientation or direction) *x* in the region of (- *π,π*] with the periodic condition. Neurons are aligned in the network according to their preferred stimuli. The neuronal connection pattern *J*( *x,x’*) is translation-invariant in the space. The network can hold a continuous family of bump-shaped stationary states. ( **B**) The stationary states of the CANN form a subspace in which the network states are neutrally stable. The subspace is illustrated as a canyon in the state space of the network. The movement of the network state along the canyon corresponds to the position shift of a bump.

The CANN is a network model for neural information representation in which stimulus information is encoded in firing patterns of neurons, corresponding to stationary states (attractors) of the network. Compared with other attractor models, such as the Hopfield network ^13^, the most prominent character of a CANN is its translation-invariant connections between neurons; that is, the connection strength between two neurons depends only on the difference between their preferred stimuli, rather than on the preferred stimulus values. This translation-invariant connection structure enables a CANN to hold a continuous family of attractors (stationary states), rather than isolated ones, with each of the attractor states encoding a stimulus value (this is where the name “continuous attractor” comes from). These states are often called bumps because of the localization of their activities in feature space. They form a submanifold of neutrally stable states in the state space of the network dynamics (see the illustration in Figure 1). This neutral stability endows a CANN with the capacity of updating its states (internal representations of stimuli) smoothly under the drive of an external input. Several mathematical formulations for CANNs have been proposed in the literature. Here, for convenience of description, we present the one whose dynamical behaviors are analytically solvable ^14, 15^, although the dynamical behaviors of many other models are similar.

Consider a one-dimensional continuous stimulus *x*, such as head-direction or orientation, encoded by an ensemble of neurons, and the value of *x* is in the range of (- *π,π*] with a periodic boundary. In the space of stimulus *x*, neurons are aligned in the network according to their preferred stimulus values. Denote *U*( *x,t*) as the synaptic input at time *t* of the neurons whose preferred stimulus is *x*, and *r*( *x,t*) the neuronal firing rate. The dynamics of *U*( *x,t*) are determined by the recurrent input from other neurons, its own relaxation, and an external input *I* ^*ext*^( *x,t*), which is written as,

$$\tau \frac{\partial U(x,t)}{\partial t}=-U(x,t)+\rho \int_{x^{\prime }}J(x,x^{\prime })r(x^{\prime },t)dx^{\prime }+I^{ext}(x,t),\;(1)$$

where *τ* is the synaptic time constant and *ρ* the neuron density. *J*( *x*, *x*′) is the interaction strength from neurons at *x*′ to neurons at *x*, and is chosen to be $J(x,x^{\prime })=J_{0}/\left(\sqrt{2\pi }a\right)exp\left[-{\left(x-x^{\prime }\right)}^{2}/2a^{2}\right],$ where the parameter *a* controls the neuronal interaction range. Note that *J*( *x*, *x*′) is a function of ( *x* – *x*′); that is, the neuronal interaction is translation-invariant in the space of neuronal preferred stimuli. The neuronal firing rate *r*( *x*, *t*) is determined by the synaptic input according to

$$r(x,t)=\frac{{\left[U(x,t)\right]}_{+}^{2}}{1+k\rho \int {\left[U(x^{\prime },t)\right]}_{+}^{2}dx^{\prime }},\;(2)$$

where [ *U*] _+_ ≡ max( *U*, 0). The neuronal firing rate first increases with the input and then saturates gradually because of divisive normalization by the total network activity. In the absence of external input and for $0<k<k_{c}\equiv \rho J_{0}^{2}/\left(8\sqrt{2\pi }a\right),$ the network holds a continuous family of stationary states, which are written as $\bar{U}(x|z)=U_{0}exp\left[-{\left(x-z\right)}^{2}/\left(4a^{2}\right)\right]$ and $\bar{r}(x|z)=r_{0}exp\left[-{\left(x-z\right)}^{2}/\left(2a^{2}\right)\right].$ These stationary states are translationally invariant and have a Gaussian-bump shape with a free parameter *z* indicating their positions.

**Figure 2.**
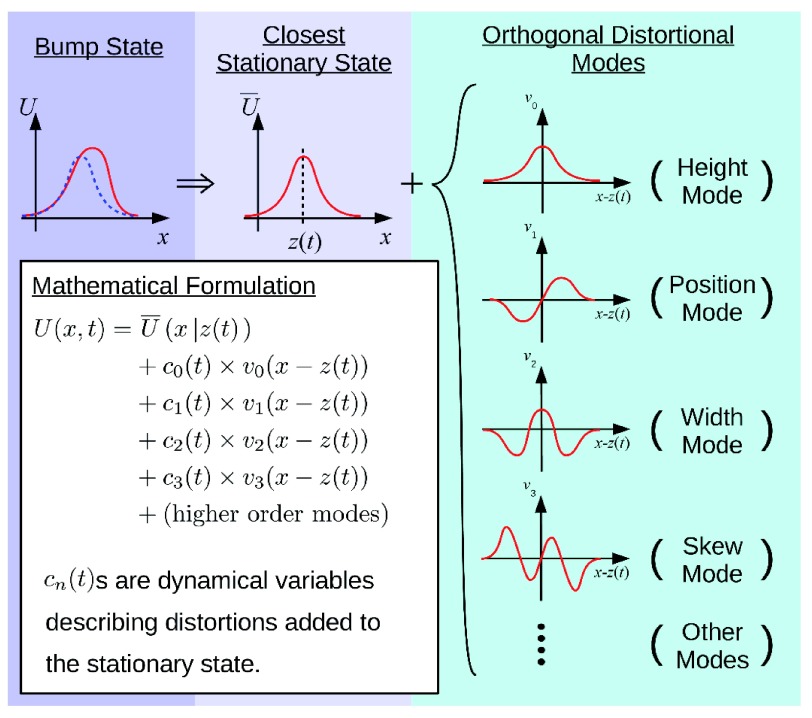
The projection method. The dynamics of a continuous attractor neural network are dominated by a few motion modes, corresponding to distortions of the bump shape in height, position, width, skewness, and so on. We can project the network dynamics on these dominating modes to simplify it significantly.

The dynamical behaviors of a CANN can be readily analyzed by a projection method ^14^ by using the property that the dynamics of a CANN are dominated by a few motion modes, which correspond to distortions of the bump shape in terms of height, position, width, skewness, and so on in the bump shape ( Figure 2). We can project the dynamics of a CANN onto these dominating modes and simplify the network dynamics significantly. Typically, by including one or two leading motion modes, the simplified dynamics are adequate to capture the main features of a CANN.

## Computational advantages of CANNs

A large volume of theoretical studies revealed many computationally appealing properties of CANNs (see, for example, 2, 3, 8, 15). Here, we present two that were recently proposed in the literature.

## CANNs for anticipative tracking

**Figure 3.**
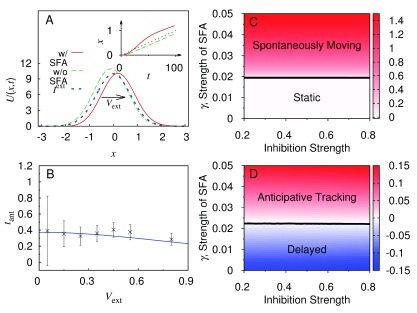
Anticipative tracking with a continuous attractor neural network. ( **A**) In the presence of spike-frequency adaptation (SFA), the network bump (red solid curve) leads the external input (blue dotted curve) moving with velocity *V*_ext_. Without SFA, the bump (green dashed curve) lags behind the external input. Inset: the positions of the bumps as a function of time when the external input starts to move at a constant velocity after *t* = 0. ( **B**) The anticipative time *t*_ant_ is approximately constant in a broad range of *V*_ext_. Symbols represent anticipative time from Goodridge and Touretzky ^22^ rescaled for comparison. ( **C**) Static and spontaneously moving phases in the space of inhibition strength $\tilde{k}\equiv k/k_{c}$ and SFA strength *γ*. The black curve indicates the phase boundary separating the static and moving phases. In the moving phase, the color code encodes the speed *V*_int_ of the spontaneously moving bump. ( **D**) Regions of delayed and anticipative tracking in the same space when there is a weak and slowly moving external input. The black curve indicates the boundary separating the delayed and anticipative tracking regions. The color code encodes the anticipative time (negative values indicate delayed time). Note the correspondence between the anticipative time and *V*_int_ in ( **C**).

Time delays are pervasive and significant in neural information processing; for example, visual signal transmitting from the retina to the primary visual cortex (V1) takes about 40 to 80 ms ^16^. If these delays are not compensated properly, our perception of a fast-moving object will lag behind its true position in the visual world significantly, impairing our vision and motor control. A CANN is able to track a moving object smoothly. However, its reaction is always lagging behind the object location because of the time needed for neuronal responses and neuronal interactions. In recent studies, Fung *et al.* ^17^ and Mi *et al.* ^18^ found that, by incorporating slow negative feedback modulation in the network dynamics, a CANN is able to achieve anticipative tracking, compensating for delays in neural systems ( Figure 3A). The negative feedback modulation can be realized by a number of mechanisms, including spike-frequency adaptation in neuronal firing ^18^, short-term depression of neuronal synapses ^19^, or negative feedback from a connected network ^20^. Since the negative feedback reduces the firing rate at the peak of the bump but is weaker at the shoulder, the tendency of the bump to move to its vicinity is enhanced. This increases its mobility, and the bump can move spontaneously when the negative feedback is sufficiently strong. Remarkably, the parameter region of spontaneous motion effectively coincides with the region of anticipative tracking ( Figures 3C and 3D). Different models relying on the asymmetric connections between neurons in a CANN were proposed to generate anticipative neural responses (see, for example, 6, 21). Here, the mechanism based on the negative feedback modulation has the advantage of realizing a constant anticipative time irrespectively of the object speed ( Figure 3B), agreeing with the experimental finding on the anticipative behavior of head-direction neurons in rodents ^22^.

## CANNs for multi-sensory information integration

**Figure 4.**
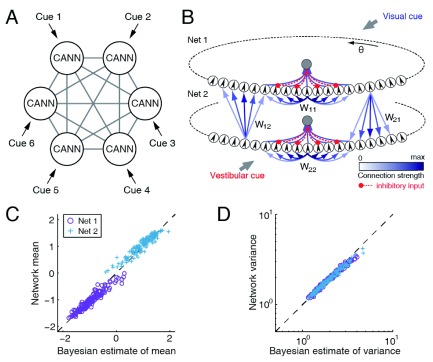
Optimal multi-sensory integration with coupled continuous attractor neural networks (CANNs). ( **A**) Multiple reciprocally coupled CANNs form a decentralized information integration system. ( **B**) An example of two-coupled CANNs for heading-direction inference with combined visual and vestibular cues. The mean ( **C**) and the variance ( **D**) of the network estimations agree with the Bayesian predictions. Adapted from 24.

The brain exploits multiple sensory modalities to gather, from different channels, as much information as possible about the surrounding environment. Psychophysical studies reveal that the brain can integrate these different sensory cues optimally to improve its perception ^23^. However, exactly how the brain achieves this remains largely unknown. In a recent study, Zhang and Wu ^24^ proposed that the brain may employ a decentralized architecture with coupled CANNs to carry out this task. In their model, multiple CANNs, each corresponding to one sensory module, are reciprocally connected with each other, and the connection strengths control the extent of integration ( Figure 4A). Mediated by reciprocal interactions, information from different cues is exchanged between sensory modules, such that global information integration is achieved at each local processor without the need for a centralized integration unit. By applying this model to the visual and vestibular cue integration for inferring heading direction, Zhang and Wu ^24^ showed that the decentralized architecture with coupled CANNs can explain a large volume of data about the integration behaviors observed in the multi-sensory experiments ( Figures 4B–4D).

## Neural signature of CANNs

**Figure 5.**
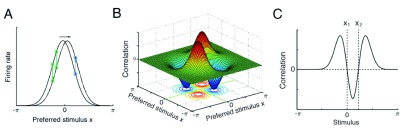
The special correlation structure associated with the unique dynamics of a continuous attractor neural network (CANN). ( **A**) The bump position-shift is the dominating motion mode of a CANN induced by input noises. Consider the true stimulus fixed at zero. Neurons at the same side of the stimulus are positively correlated (e.g. green ones), whereas neurons on different sides of the stimulus are negatively correlated (e.g. green versus blue ones). ( **B**) When the true stimulus is fixed at a constant value (e.g. zero), the correlations between all neuron pairs in a CANN display an anti-symmetric structure. ( **C**) When the stimulus value varies, the correlation between a fixed neuron pair with a typical separation of the bump width displays an M-shaped structure.

The key structure of a CANN is the translation invariance of the connections between neurons. Limited by experimental techniques, we are still unable to confirm the existence of such a connection pattern of synapses in real neural systems. Nevertheless, we can validate the existence of a CANN by measuring its unique dynamical features. One such feature is the anti-symmetric, or the M-shaped, correlation between neuronal responses ^15, 25^. The underlying cause is intuitively understandable. The dynamics of a CANN are dominated by the position shift of a bump under the drive of noisy inputs. Thus, in response to a stimulus corrupted with noise, neurons whose preferred stimuli are on the same side of the true stimulus (i.e. they are both larger or both smaller than the stimulus value, as illustrated in Figure 5A) will increase or decrease their responses concurrently with the fluctuations of the bump position, leading to a positive correlation, whereas for neurons whose preferred stimuli are on different sides of the stimulus, their response fluctuations are negatively correlated ( Figure 5B). Alternatively, we can measure the correlations of firing rates between a pair of neurons with a typical separation of the bump width in a CANN by varying the stimulus value systematically. In such a case, we obtain the M-shaped correlation over the stimulus value space: when the stimulus value is in between the preferred stimuli of two neurons, the neuronal responses are negatively correlated; otherwise, they are positively correlated ( Figure 5C).

Notably, the M-shaped correlation between a neuron pair in the cortex was confirmed in recent experiments. In the study, Wimmer *et al.* ^26^ used multiple electrodes to record the activities of neurons in the prefrontal cortex (PFC) of monkeys when they were performing a working memory task. The monkeys were firstly presented with a stimulus appearing randomly in one of eight possible directions, and then the monkeys needed to memorize the stimulus location during a delay period when the stimulus was off. Wimmer *et al.* found that the dynamics of a CANN can well explain the behaviors of the monkey and that the correlation between PFC neurons in the delay period is M-shaped. In another study, Ponce-Alvarez *et al.* ^25^ measured the correlation between a neuron pair in the middle temporal area when monkeys were presented with moving grating or plaid, and also found the M-shaped structure.

## Beyond simple features

In addition to the aforementioned simple, straightforward features of objects, such as orientation, direction, and spatial location, experimental data suggest that the brain may use CANNs to process less directly perceivable features. For example, in an experimental study, Logothetis *et al.* ^27^ found that, after training, neurons in the inferior temporal cortex of the monkey’s brain displayed strong selectivity to the view angle of an object and that the neuronal tuning functions were of the bell shape and were aligned to cover the space of view angles, similar to the structure of a CANN. Furthermore, the brain may use CANNs to process “complicated” features. In recent work, Mante *et al.* ^28^ studied the dynamical properties of neuronal responses in the PFC when monkeys executed a context-dependent choice task, in which the monkeys made a left or right saccade depending on a flexible context cue, which was either direction of motion or color. Interestingly, the authors found that although individual neurons’ responses exhibited intractable complexity, the neural responses at the population level follow a low-dimensional trajectory embedded in the high-dimensional space. Mante *et al.* further trained a network model to interpret the experimental finding and found that the CANN structure automatically emerged in the trained network: the network held a set of stationary states forming the canyon of a one-dimensional CANN, integration of cue evidence was implemented in the model as movement along the canyon (approximately), and different strengths of sensory inputs led to different stationary states in the canyon. The CANN model well reproduced the experimental data, indicating that PFC neurons may exploit the CANN structure to encode the subject value of evidence/confidence in decision making.

## Further aspects

In conclusion, the accumulated facts, including the computationally appealing properties, many successful examples in modeling brain functions, and the new supporting experimental data, suggest that CANNs may serve as a canonical model for information representation in neural systems. Nevertheless, there is still a lot of work to do to validate this hypothesis. In experiments, the vast development of imaging techniques will eventually give us direct evidence of whether neuronal synapses are translation-invariant in a feature space and, if they are, where in the brain and to what extent. In theory, as implied by the above-reviewed work, we should explore deeply the functional roles of CANNs, including how CANNs contribute to the information exchanges constantly happening between cortical areas, how the CANN structure emerges automatically via either supervised or unsupervised learning in a given computational task, and how a CANN, which has the capacity of encoding complex, non-trivial features of external inputs (as suggested by the work of Mante *et al.*), contributes to the categorization of objects or formulation of concepts. Overall, these studies will not only enhance our understanding of the principles of neural information processing but also reveal more computational advantages of CANNs which are useful for developing brain-inspired computation algorithms.
